# Insiders’ Insight: Discrimination against Indigenous Peoples through the Eyes of Health Care Professionals

**DOI:** 10.1007/s40615-018-0495-9

**Published:** 2018-05-07

**Authors:** Lloy Wylie, Stephanie McConkey

**Affiliations:** 1 Departments of Pathology, Psychiatry, Anthropology and Health Sciences, Schulich Interfaculty Program in Public Health, Western Centre for Public Health and Family Medicine, Western University, 4101 - 1465 Richmond St., London, ON N6G 2M1 Canada; 2 Department of Epidemiology and Biostatistics, Western University, London, ON N6G 2M1 Canada

**Keywords:** Discrimination, Health services, Indigenous peoples, Aboriginal people

## Abstract

Discrimination in the health care system has a direct negative impact on health and wellbeing. Experiences of discrimination are considered a root cause for the health inequalities that exist among Indigenous peoples. Experiences of discrimination are commonplace, with patients noting abusive treatment, stereotyping, and a lack of quality in the care provided, which discourage Indigenous people from accessing care. This research project examined the perspectives of health care providers and decision-makers to identify what challenges they see facing Indigenous patients and families when accessing health services in a large city in southern Ontario. Discrimination against Indigenous people was identified as major challenges by respondents, noting that it is widespread. This paper discusses the three key discrimination subthemes that were identified, including an unwelcoming environment, stereotyping and stigma, and practice informed by racism. These findings point to the conclusion that in order to improve health care access for Indigenous peoples, we need to go beyond simply making health services more welcoming and inclusive. Practice norms shaped by biases informed by discrimination against Indigenous people are widespread and compromise standards of care. Therefore, the problem needs to be addressed throughout the health care system as part of a quality improvement strategy. This will require not only a significant shift in the attitudes, knowledge, and skills of health care providers, but also the establishment of accountabilities for health care organizations to ensure equitable health services for Indigenous peoples.

## Introduction

Indigenous patients’ experiences of disrespectful treatment by health care providers remain a significant barrier to health care access [1]. Research shows that discriminatory behavior is still present in the Canadian health care system today [1–4], and stories of negative experiences of Indigenous patients are seen in the headlines of Canadian media [5–7]. In some cases, the lack of consideration for the concerns of Indigenous patients has resulted in detrimental, if not fatal, consequences [8].

Indigenous peoples’ narratives about poor treatment by health care providers draw clear conclusions that racism was a factor in those interactions. Stories of misdiagnoses of late-stage cancers to assumptions of drunkenness of patients with acute health episodes demonstrate that the health concerns of Indigenous people are often disregarded by health care professionals. The story of Brian Sinclair, who died in the Emergency Room in 2008, is a case in point. His physician had referred him to the emergency room as he had a blocked catheter. Health care workers assumed that Sinclair was a drunk, poor, and homeless Indigenous man seeking shelter, and therefore, he was never triaged into the system. He waited 34 h in the waiting room and was pronounced dead when a physician finally decided to see him [6–8].

This case from over a decade ago demonstrates the significant consequences of racism and stereotyping on the health outcomes of Indigenous peoples in Canada. More recent research had demonstrated that such experiences are not an anomaly. The Canadian health care system is often a place of negative experiences for Indigenous peoples, making them reluctant to access services [ 2, 3, 9], and creating significant barriers to health equity [1–4]. Many Indigenous people have admitted their reluctance to continue accessing health services after their experience with discrimination in the health care system. Some patients avoid services altogether [2, 3, 9], while others prepare to be mistreated by health care providers prior to accessing services [3]. These and other instances have been brought forward by Indigenous peoples and organizations across Canada and have renewed the call for Indigenous health research to focus on discrimination in health care systems [3, 10]. The fact that these negative experiences remain so widespread should be a societal concern, as it demonstrates how our health care system is failing the most vulnerable. This is not just a concern about access; it is a matter of life and death, as the case of Brian Sinclair demonstrates. If Canada is genuinely on a path of reconciliation in the wake of the recent Truth and Reconciliation Commission in 2015, the time for change is well overdue.

This study, *Engaging for Change*, explored the attitudes, knowledge, and practices of health care providers around working with Indigenous patients. The purpose of collecting this information was to understand the current state of these attributes among health care providers, as part of the information needed to uncover what shapes the experiences of Indigenous people with health care services in a large city in southern Ontario. Having knowledge of these attributes can contribute to informing strategies for changing these norms in ways that will improve Indigenous peoples’ health care experiences. This paper presents the findings on health care providers and decision-makers’ perspectives on discrimination towards Indigenous people in the health care system. By gaining insight into what care providers think, know, and do in the health care setting can help identify systemic challenges and identify opportunities for targeted improvement initiatives to address discriminatory practices and increase the uptake of culturally safe care.

## Background

Research in the USA has extensively examined the relationship between race and health, demonstrating that people who experience racism have poorer mental and physical health outcomes [3, 11], therefore concluding that discrimination is a social determinant of health that has a significant impact on minority populations [12, 13]. More specifically, studies on racism in health care have noted that implicit bias of health care providers result in differential clinical treatments and decisions, leading to lower quality care for racialized groups [14, 15]. Physicians in the USA have reinforced that differential treatment based on race does exist in the health care system, as they acknowledged that they had more positive associations with their non-racialized patients when compared to their racialized patients [8, 11].

Discrimination against Indigenous peoples has unique historical roots in colonialism [16]. As Tuhiwai Smith (1999) has argued, colonialism required “the imposition of Western authority over all aspects of indigenous knowledge, languages and cultures” [17]. Indigenous peoples were subjected to “paternalistic and racist policies and legislation; … necessary conditions which had to be met if Indigenous peoples wanted to become citizens (of their own lands)” [17]. This early subjugation demanded that Indigenous ways of knowing and being were characterized as primitive and “savage.” As Henry et al. (2000) have argued, the colonization of Canada “relied on a belief system that judged the original inhabitants to be inferior,” relying on racist ideologies of Social Darwinism [18]. This constructed inferiority of Indigenous peoples continues to influence deeply held stereotypes that perpetuate discrimination against Indigenous peoples today.

Discrimination is one of the root causes of the health inequities that exist between Indigenous and non-Indigenous peoples [19]. Research has demonstrated that experiences of discrimination is commonplace among Indigenous peoples, with patients noting abusive treatment, stereotyping, and a lack of quality in the care provided [1], which discourage Indigenous people from accessing care [2, 3, 8]. Indigenous patients have expressed that they are treated like intruders in the health care system [1], not welcome and provided substandard care. Recent studies have noted that Indigenous peoples often endure experiences of racism within the health care system [3, 20]. Furthermore, one study that examined experiences of Indigenous peoples in the health care system demonstrated poorer mental, physical, and behavioral health among those who were subjected to racist treatment, and that 1 in 3 participants experienced a microaggression (a casual derogatory remark or action) against them by a health care professional [19]. The recent findings of Allan and Smylie (2015) have demonstrated that the problems that were identified by researchers over the past 10 to 15 years have not substantially changed; as the title of their study suggests, First Peoples continue to get second-class health care [3]^.^

Growing recognition of the health challenges Indigenous people experience in Canada through the national dialog about the Truth and Reconciliation Commission has created a broader sentiment to address these challenges. The calls to action from the Truth and Reconciliation Commission [21] provide recommendations for strategies to address these systemic challenges. In addition, recent mandates from Health Quality Ontario [22] expect hospitals to have an equity measure, which has created greater incentives to address the access barriers that Indigenous people often face in health care. Although more people are recognizing the health inequities faced by Indigenous people, there are many gaps in the knowledge, skills, and attitudes of health care providers to adequately address the problem, including attitudes informed by anti-Indigenous racism. This opening created by the national dialog in the wake of the Truth and Reconciliation Commission is an opportunity to reflect on the anti-Indigenous racism, as it demands attention to the experiences of Indigenous peoples [23].

## Research Aims

The purpose of this research project was to examine ways to improve Indigenous patients’ experiences when accessing health care. Unlike in British Columbia where the recognition of widespread challenges and new intergovernmental agreements have put Indigenous health on the agenda [24, 25], attention to Indigenous peoples’ experiences in health care seemed absent from the health care dialog in Ontario in 2015. Compared with the BC experience, where all health authorities have been mandated to take responsibility for improving First Nations peoples experiences with their health care organizations [25], there seemed to be a real lack of concern among health care providers and educators about Indigenous health equity. Some even stated that equity was not within the scope of responsibility of hospital services. When raising issues on Indigenous health inequity, the authors were faced with confused stares and a declaration that as a hospital, “health equity was not really our job.”

The cases and research presented in the beginning of this paper brought forward perspectives from Indigenous patients about their experiences in health care. Rather than choosing to speak with Indigenous patients, given that others had already brought forward those experiences, this research chose to initially focus on the perspectives from health care providers. This research project, *Engaging for Change*, aimed to understand if care providers and decision-makers were aware of the problems and barriers that Indigenous people face regarding health care. The assumption behind this choice was that a first step in developing a plan to change practice in health care organizations was to find out if people working within those organizations were ready to engage in that change process. Priority health service areas were determined through discussions with Indigenous health leaders, and included emergency, diabetes, maternity, cancer, and mental health. Research respondents were selected from those service areas.

We carried out a baseline analysis of the current state of cultural competency and safety skills among health care providers working in a large city in southern Ontario. Through exploring attitudes, knowledge, and skills of health care personnel across the five priority health service areas, this study sought to identify promising practices, challenges, and gaps in the provision and continuity of health care for Indigenous peoples. The purpose of this research is to provide an evidence base to inform improvements in services for Indigenous peoples across hospital- and community-based health services, by identifying the current challenges and opportunities for change among health care providers, decision-makers, and organizations. This paper provides the findings related to the barriers shaped by discrimination against Indigenous peoples when they access health care services.

## Methods

Priority research areas and topics were identified through a comprehensive literature review and discussions with community leaders in Indigenous health in the city and surrounding area. Ethical approval was granted by the ethics boards of the health sciences center and the university. A list of participants was developed that included a selection of service providers and decision-makers in emergency, mental health, maternity, diabetes, and cancer-related services within the city and the surrounding area. Interviews and focus groups with health care workers were carried out once written consent was obtained. Qualitative one-on-one interviews (*n* = 21) and focus groups (*n* = 2) were completed from August 2015 to June 2016, totaling 25 respondents. Interview and focus group sessions were approximately 1 to 1.5 h in duration. Physicians, nurses, social workers, patient navigators, patient experience specialists, and departmental directors/leaders were interviewed to understand health care workers’ perspectives and experiences with Indigenous patients. Semi-structured interviews among participants included 20 in-depth questions to understand their perspectives of Indigenous health access barriers, experiences providing care to Indigenous peoples, and understanding of organizational structures (i.e., policies and protocols) for facilitating inclusion of Indigenous-specific needs. Participants were also asked about their awareness of local community health services and resources for Indigenous people in the city and the surrounding area.

For purposes of this study, Indigenous peoples were defined as the First Nations, Inuit, and Métis peoples of Canada. Interviewees were asked to reflect on the experiences of their patients they knew or perceived were Indigenous. Indigenous and Aboriginal will be used interchangeably to describe the First Nations, Inuit, and Métis peoples of Canada throughout this paper, to ensure that the respondent quotes are presented verbatim.

All interviews and focus groups were voice recorded, transcribed verbatim, and coded using NVIVO 10 Software. Discrimination was a common theme that emerged throughout the analysis, and relevant quotes were identified across multiple thematic nodes including cultural barriers for patients, patient/physician relationships, perceived discrimination by patients, perceived discrimination by care providers, systemic discrimination, stigma, stereotyping, discriminatory beliefs, and racism. Quotations were extracted from the data and organized into subthemes around discrimination. This paper explores attitudes and practices of discrimination towards Indigenous people as identified by people working within the health care system in a large city in southern Ontario.

## Results

The results from this study demonstrate that experiences of discrimination are systemic in the health care system. This is not just from the perspective of Indigenous patients, but also recognized by those working within the health care system. Respondents expressed that they have witnessed systemic discrimination and racist practices against Indigenous people that are influenced by stereotypes, societal misconceptions, and poor cultural competence of health care workers. Respondents identified a range of experiences of racist or exclusionary practices, from the subtler unwelcoming nature of the hospital environment to more concerning examples of compromised care. The results are presented along this spectrum, organized into three main discrimination subthemes, including an unwelcoming environment, stereotyping and stigma, and practice informed by racism.

### Unwelcoming Environment

A common theme among respondents was that health service organizations are an unwelcoming environment for Indigenous patients and families. A few respondents acknowledged that Indigenous peoples may not feel welcome in the health care system due to past negative experiences with health care organizations and health care workers, or as a result of their experience with residential schools and Indian hospitals. These negative experiences are seen to contribute to the overall hesitancy to access mainstream health services. One health care leader explained,

I would say that they have a perception that they are perhaps as not well cared for or as respected when they come in to the facility. Maybe that is a reluctance for them to come and they seek treatment in other places they feel more comfortable with (A12L).

A number of respondents noted that there are times when Indigenous patients come in with expectations of poor treatment, which sets the stage for a challenging interaction with care providers. One respondent explained that they (as a physician) sometimes become defensive when an interaction with an Indigenous patient is not going well. Specifically, they shared,

I don’t think we are the most welcoming environment. So I think [Aboriginal patients] come in with a preconceived notion that they are going to be marginalized or viewed differently or kind of not treated as well as they should be or viewed differently. I think some Aboriginal people come with a preconceived notion which on an interaction is not a good way to start because they are already on the defensive and it puts you on the defensive. And it can feed on itself (A14P).

When Indigenous patients expect to be treated poorly when they access health services due to past experiences of discrimination [3], they may be more likely to question the quality of care they receive. One respondent provided an example of a time that an Indigenous patient believed that a regular delay in the emergency room a result of racism:

I remember one lady a long time ago, maybe 12 years ago, she was waiting in the emergency for a long time and she made a comment out loud about the wait time and she said that her wait time was discriminatory because of her Aboriginal status. But the staff was able to explain to her about the triage system and how it worked and that the wait time was not related to that at all. It’s hard to know if the patient truly believed that or not, but at least she appeared to believe it. That is the only thing that I have experienced and I wouldn’t even construe it as negative. I could perceive that that patient had a negative perspective of how things were going in the emergency department (A01P).

General frustrations with health care service were also expressed through accusations of discrimination. Other respondents noted similar issues where patients expressed concerns of discrimination they felt were unfounded.

I saw a patient yesterday who has schizophrenia and personality disorder. Actually his schizophrenia is under excellent control. But he is dissatisfied with a variety of things, including the level of service. He wants lifts all the time, and when he doesn’t get them, he gets annoyed. He’s under the public guardian and trustee to assess managing his finances and all that. So we were negotiating around that. But often, he gets frustrated and accuses me of being biased towards him. Brings up residential schools, that this is a consequence of further insult of the residential school system. It’s a diversionary issue which doesn’t help with anything…But that is a negative experience. I think it takes away from the development of relationships. Unfortunately, that isn’t the only person where I have had that experience where it comes to a difficulty, this issue is brought up. Sort of a scolding (H14P).

Another respondent provided a similar case:

We have a gentleman now who is on the transplant list and he is going to be a challenging transplant because he has a lot of anti-bodies…He’s questioning if there is any element of discrimination of why he’s not getting his transplant (A07P).

These examples demonstrate that due to the lack of trust and good relationships that Indigenous people have with health care providers and the health system in general, poor communication about processes in health care can easily be misconstrued.

Although the providers quoted above express that the defensiveness of their Indigenous patients are unfounded, it is common for the “forces of colonization to go unrecognized in the health care system” [26]. Despite this general blindness to institutional oppression, most of the respondents were clear that the defensiveness among Indigenous patients was a natural response to the judgmental and often hostile reception Indigenous people experience in the health care setting. Many respondents were clear that the hospital environment is indeed unwelcoming and inflexible in many ways, and particularly for Indigenous people. As one respondent noted: “we do have challenges accepting the cultural differences and I hear that from staff” (A22L). Another respondent expressed that “they have to fit in to this big melting pot of a system that we have with very little consideration by the non-Aboriginal population as to what works best for that cultural population of people. I think that’s a huge thing” (A18L). Of particular note is that multiple respondents referred to the lack of consideration for different approaches to health, with a strong conviction of the supremacy of a western approach. As one respondent noted,

I think that we also do a very poor job of…accepting non-Western medical therapies, so if anyone talks about naturopathic or holistic healing or whatever other approaches… whether you say it explicitly or apply it in your actions, it is really kind of frowned upon and thought of as a lesser way to care…I would say that of almost every trainee from the medical school (A13P).

The ability to ensure that cultural preferences are incorporated into care is seen as a particular challenge in some areas of hospital services. As one respondent stated:

I think one of the other barriers is they are intolerant to the welcoming of other healing practices. You know, you are in the ICU and you know…everything is so tightly controlled and if you want to add to that, some other cultural healing practices or have a healer come in, that, I mean it certainly has happened and it does happen and I think people try, but it is enormously difficult in such a controlled environment…Once you are in hospital it is extremely difficult…I think particularly challenging in the ICU, although I have had experiences of having physician and pharmacy staff getting very involved in trying to support that. But, it is still with a permission aspect, it is still a very controlled environment and the control is by our health care system (A20S).

Overall, it was clear that respondents were able to recognize a disconnect between Indigenous peoples and conventional health care providers, particularly emphasizing organizational norms that are not open to adapting to the needs or preferences of Indigenous people. These examples demonstrate that hospitals and other health care settings are often an unwelcoming, inflexible environment for Indigenous peoples. This situation often creates negative expectations, where Indigenous people anticipate and prepare for negative behaviors from health care providers prior to accessing services [3]. Furthermore, it is evident that there are significant communication barriers between health care workers and Indigenous patients. Although defensiveness was present among some of the care providers in our study, who emphasized that they treat everyone the same, more often the attitude among the respondents was one of uncertainty on how to engage in meaningful conversations without offending patients. Improving the confidence of care providers to engage in meaningful and non-judgmental dialog with Indigenous patients should be an important part of any training to improve their communication skills. If trust is not gained and relationships of mutual respect are not built as a first step, negative interactions continue to perpetuate misunderstandings and an unwelcoming environment, whether it is intentional or not.

### Stereotyping and Stigma

A number of respondents acknowledged their lack of understanding and knowledge of Indigenous issues, culture, and medicinal practices. Instead, they noted that views on Indigenous people are informed by stereotypes perpetuated in the media. One respondent emphasized that systemic discrimination is a significant issue in the health care system that shapes health outcomes. They explained,

There has been a lot of stereotyping and bias. To me, that is just one issue that the report, 'First People, Second Class Treatment' is that racism has been identified as something that is a social determinant of health and that has a very strong influence on outcomes for Indigenous people. I would say that it’s universal. And not just here. And its everywhere. That is one core issue (H06L).

This response suggests that discrimination is a key social determinant of health for Indigenous people. Health care workers underestimate the negative consequences and detrimental effects of discrimination on the health and wellbeing of Indigenous peoples. A respondent shared their beliefs about how Indigenous patients continue to be stereotyped in the health care system and acknowledged the lack of cultural consideration Indigenous patients receive from health care workers, noting that there are

stereotypes that continue to exist among generations of non Aboriginals as to what Aboriginal folks’ lifestyles are. Their upbringing, their level of education; dozens and dozens of these preconceived ideas or assumptions. Been validated? I don't think they have been validated personally, but maybe through the media which paints everyone with a very large brush as you know and probably not doing any justice to the Aboriginal person (A18L).

This is a very powerful statement that explains how ideas from the past and current media discourses reinforce common misconceptions about Indigenous peoples. These misconceptions influence non-Indigenous people’s behaviour towards and perceptions about Indigenous peoples, in an overwhelmingly negative way. Another respondent admitted to widespread “judgment in the system, we judge that they’re idiots when they’re not (A07P).” A patient support staff also shared feedback they had received from Indigenous patients regarding their experience using the health care system. They noted “the themes that we were hearing from the different experiences were quite negative and they often involved a level of discrimination, of stereotyping. People who really felt uncomfortable in their health care setting. And unsupported” (H06L).

Some staff felt that there were strong tendencies of prejudicial and racist attitudes among health care providers in this southern Ontario city:

A problem [here] is stigmatization. I find it very conservative, I come from up north. I mean, I worked on reserve for years and it, I mean it was just a part of [the town], you didn’t really see that visible minority like you see here. People just…I don’t know; it is just totally different here. I just find the whole atmosphere different here. In the north, we are just like, here is your neighbour, and away you go. Here, its just like, “ok, well that person looks quite different.” So I think that might be a challenge here (H17L).

Although not all health care workers are aware of the frequency or depth of systemic discrimination that is present in today’s health care system, respondents noted that discrimination was an issue that needs to be addressed. As one respondent acknowledged, “I think there is still a lot of racism and discrimination and I think that is something that they unfortunately they still face” (AB10P). Another respondent shared:

People stereotype them. ‘oh they are like this, they don’t take care of themselves’ is kind of the picture I get from other staff…I have seen that personally. It could be a discrimination or lack of understanding, cross-cultural understanding, how these people are suffering, why and how we can help them (AH11P).

Negative stereotypes held by health care providers were identified as a significant problem for Indigenous people accessing health care services. Respondents felt that these prejudicial beliefs among many of their fellow health care staff significantly undermined their ability to provide good care for Indigenous people.

### Practice Informed by Racism

Discrimination influences health care providers’ interactions with patients. Respondents noted that physicians commonly blame Indigenous individuals for their health status. Care providers are often unaware of the social determinants of health that have led to poor health outcomes among racialized populations, partly due to the lack of research on this area [27]. A respondent identified this lack of knowledge around the determinants of Indigenous peoples’ health, noting that “because of things that are more endemic in their populations such as drug and alcohol abuse, which can be stigmatized but not acknowledged in terms of why its come about, can be a problem (A09P-FG).” This respondent clearly stated that many care providers believe that health issues experienced by Indigenous patients are simply because they do not take care of themselves. Health care providers often completely disregard the social determinants of health as explanations for ill health among Indigenous patients and demonstrate a lack of understanding of the effects of colonization. They also fail to understand that such negative historical practices have led to a number of systemic challenges such as poverty, abuse, and cultural genocide [28], which have a detrimental effect on Indigenous people’s health and wellbeing. Health care workers that ignore these systemic challenges are unable to provide adequate care for Indigenous patients because they do not understand their specific needs or how to help and support them.

One respondent identified a case where the physician’s reaction to an Indigenous patient was clearly discriminatory. They felt that “the response is also informed by racism. Someone else might have the same behaviour and you wouldn’t get that level of response (A20S-FG).” This statement suggests that Indigenous peoples face unequal treatment in the health care system, which may be informed by discrimination and racist attitudes of health care workers.

One respondent shared that health care workers carry common preconceived notions and do not take time to understand their Indigenous patients beyond those common misconceptions. Specifically, they felt that providers’ attitudes towards their Indigenous patients are characterized by “not understanding; not taking the time; labelling (A18L).” Indigenous patients are getting labeled based on discriminatory stereotypes, which in turn influences the kind of care that they receive from health care providers.

The vast majority of comments about the practices informed by discriminatory attitudes spoke about the hesitation to prescribe pain medication. Respondents reported incidences where they witnessed care providers’ reluctance to prescribe pain medications due to common misconceptions about Indigenous peoples as drug abusers. One respondent acknowledged the general lack of standards for pain medication prescriptions, based both on poor training and perceptions about Indigenous peoples and addiction:

It’s my bias and I put it out there…My perception is that it’s a bigger issue with the use of narcotics and I think to be honest, we’re poorly trained…I’m not a social worker, I know nothing of past and dealing with emotions and pain from bad experiences from the past, other than I realize it’s an issue. I don’t delve into that because you wouldn’t want me to. Probably get myself into a black hole that I couldn’t get out of. We do have a social worker…and I think they’re better trained at that. I think there’s a whole lot of stuff that doesn’t get unpacked and where that leads to pain and blah blah, that’s too much for me. That’s an excuse and it’s too much for me to really carry on about because I’m not trained to do those conversations…We’re either giving too much or not enough, and mine is probably not enough. I don’t prescribe narcotics unless I have some kind of clear guideline and it can be very clear, including screening urine (A07P).

The respondent acknowledged their assumption that Indigenous peoples are drug seekers, using emotional pain as an excuse. Although these assumptions informed by stereotypes led to possible under prescribing of pain medications, the respondent noted that better training is needed to ensure the safe use of narcotics for pain management among all patients.

Another respondent acknowledged that there is a system-wide belief that Indigenous peoples misuse pain medications, and as a result, Indigenous peoples are not provided adequate pain medication. One physician noted,

I would think there are, that on our side some frequency of notions that they are all drug seekers. Which clearly isn’t true. But I think that if you lined up 10 patients with the same condition and how much pain meds would you give this person if they were Aboriginal, I would bet less, because the fear would be that they are there looking for pain meds to abuse or sell (A04P).

A patient support worker acknowledged that racism and discrimination are evident in the health care system surrounding pain medications. They explained that the assumption that Indigenous people are drug abusers was widespread:

In a lot of the charts, there is always something written, suspect abuse, suspect abuse, suspect abuse…I thought, is everyone suspected of alcohol abuse and drug abuse?...A lot of the advocating that I do is, they need pain meds, they need pain meds, they need pain meds. It seems like the physicians or the medical staff seem reluctant to give it them. I have even had people who were diagnosed with cancer throughout their bones and…it’s the most excruciating form of cancer and this particular patient was prescribed Percocets…I thought Percocets were for more of a sore back or you tore a ligament or something. But not for bone cancer. So she was taking like 10 of them a day, 10 or 12 a day. Isn’t that too many pills? Can’t you give her something else that is going to work more effectively than taking 3 or 4 percocets, 4 or 3 times a day? (H05S).

This response suggests that the stereotypes that care providers have about Indigenous people influences their clinical decision making. Indigenous people are regularly being labeled as drug abusers, without an open conversation with the patient and without taking their medical history into consideration. Indigenous peoples are being denied access to necessary pain medications as a result. This is a clear example of the link between discrimination and the provision of inadequate care for Indigenous peoples.

## Discussion

This study has demonstrated a range of challenges that Indigenous people face that stem from discriminatory attitudes and behaviors of staff in the health care system. The health care providers that shared their experiences and insights with us were forthright that discrimination shaped the experiences of all Indigenous people accessing care in their organizations. The lack of a welcoming environment leads to misunderstandings when there are legitimate service delays and complications. It also sets the stage for poor interactions, preventing positive relationship and trust building between Indigenous patients and families with health care providers. This unwelcoming environment was also seen as a significant reason why Indigenous people avoid accessing mainstream health care services. The unwelcoming nature of health services is created by the assumptions that health care providers make based on stereotypes formulated through a racist discourse played out in the media again and again. These poor attitudes in turn shape the care and treatment decisions made by health care providers. Some care providers were aware of this bias, while others noted that these attitudes were so normalized in the system that many people are unaware of the problem.

Discrimination can be seen as preventing access to health services, which research has shown results in a high number of Indigenous patients receiving late diagnoses. Many times these patients are provided a disease diagnosis at a stage that is untreatable [14]. This is a significant problem that worsens the health status of a population group that is already suffering a considerably higher burden of disease across a range of health conditions [2]. This demonstrates that the Canadian health care system we tout as universally accessible, is actually perpetuating access barriers for Indigenous peoples. Attitudes based on stereotypes and stigmatization of Indigenous people are shaping practice in ways that compromise care for this entire population.

Although we know that care providers often underestimate the challenges faced by Indigenous peoples in health care [16], this study shows that it is such a widespread concern that even people who state they are unaware of many of the challenges Indigenous people face witness widespread discrimination within their own organizations [29]. Therefore, to improve health care access for Indigenous peoples, there are a range of issues that need to addressed, including discrimination and lack of knowledge about Indigenous peoples that lead to negative stereotyping. This demonstrates that the challenges in accessing appropriate and necessary health services for Indigenous peoples are uniquely shaped by discrimination that is manifested in a myriad of ways when an Indigenous person walks into a health care facility. This study validates these concerns that have been brought forward by Indigenous patients and families, as it demonstrates that people working within the system know that experiences of discrimination are commonplace and therefore require a system-wide response.

This study has shown that there are serious concerns that health care practices are being shaped by prejudice against Indigenous people, rather than by evidence-based, patient-centered care, which is a common mandate of health care organizations. Health service organizations need to address the discriminatory bias that their staff bring to interactions with Indigenous patients and families. This includes developing ways to rewrite the narrative, based on respectful relationships with Indigenous peoples and communities. Such relationships would require health care professionals to be open and non-judgmental about Indigenous patients’ health conditions and health care preferences, rather than just simply making assumptions and imposing the health care practices of mainstream services [1, 9, 30]. Unless discrimination is taken on systemically through the development of protocols based on principles of cultural safety that ensure respectful engagement with all patients and families, health care organizations will continue to perpetuate access barriers for Indigenous people.

To address this violation of Indigenous peoples’ rights to accessible health care, health facilities need to ensure that practices are based on respectful interactions with Indigenous patients and families, which ensure quality care that attends to their specific needs, while meeting high standards of practice. Providing a safe and welcoming environment for Indigenous peoples is key for working through past traumas resulting from colonial practices and to redress negative experiences with health care. This requires a significant shift in the attitudes, knowledge, and practice of health care providers who work with Indigenous peoples. In line with the recommendations of the Truth and Reconciliation Commission (2015), this training needs to be taken up by the educational institutions who train future health care providers, specifically to ensure that students are enrolled in a course that addresses Indigenous health issues. What this paper has demonstrated is that although background training may be valuable, the organizational culture within health care institutions carries a strong weight of the past, normalizing colonial practices that reinforce the inferiority of Indigenous peoples and practices. Effectively challenging and changing this deep-seated organizational culture within health care institutions will require ongoing training and vigilance to ensure new norms of equity and characterize the ethos of our health care institutions.
